# Age, growth, reproduction and management of Southwestern Atlantic’s largest and endangered herbivorous reef fish, *Scarus trispinosus* Valenciennes, 1840

**DOI:** 10.7717/peerj.7459

**Published:** 2019-08-30

**Authors:** Matheus O. Freitas, Marília Previero, Jonas R. Leite, Ronaldo B. Francini-Filho, Carolina V. Minte-Vera, Rodrigo L. Moura

**Affiliations:** 1 Programa de Pós Graduação em Engenharia Ambiental, Universidade Federal do Paraná, Curitiba, Paraná, Brazil; 2 Núcleo de Pesquisas em Limnologia, Ictiologia e Aquicultura, Universidade Estadual de Maringá, Maringá, Paraná, Brazil; 3 Instituto de Biologia and SAGE/COPPE, Universidade Federal do Rio de Janeiro, Rio de Janeiro, Rio de Janeiro, Brazil; 4 Departamento de Engenharia e Meio Ambiente, Universidade Federal da Paraíba, Rio Tinto, Paraiba, Brazil; 5 Inter-American Tropical Tuna Commission, La Jolla, CA, USA

**Keywords:** Labridae, Scarinae, Fisheries management, Abrolhos, Brazil, Marine Protected Areas, Parrotfishes, Artisanal fisheries, Greenbeack parrotfish

## Abstract

The Brazilian-endemic greenbeack parrotfish, *Scarus trispinosus* Valenciennes, 1840, is the largest herbivorous reef fish in the South Atlantic. Following the sharp decline of large carnivorous reef fishes, parrotfishes (Labridae: Scarinae) were progressively targeted by commercial fisheries in Brazil, resulting in a global population decline of 50% for *S. trispinosus*. Most of its remnant population is concentrated in the Abrolhos Bank, where the present study was conducted. We present novel information on age, growth and the reproductive cycle of *S. trispinosus*, based on 814 individuals obtained from commercial fisheries’ landings and scientific collections, between 2010 and 2013. Sex ratio was biased toward females (1:8), and spawning occurred year-round with discrete peaks in February-March and June-December. Increment analysis indicated annual deposition of growth rings in otoliths, which presented 1–22 rings. The asymptotic length at which growth is zero (*L*_∞_) was estimated from a Bayesian logistic regression at 85.28 cm, growth rate (*K*) at 0.14 year^−1^, and the theoretical age at zero size (*t*_0_) at 0.16. Subregional demographic structuring was detected, with predominance of slower-growing individuals in shallower inshore reefs and predominance of faster-growing and older individuals in deeper offshore sites. We demonstrate that *S. trispinosus* is highly vulnerable to over-exploitation due to its large size, long live and slow-growth, and review the management measures proposed since its Red List assessment in 2012.

## Introduction

Parrotfishes play key ecological roles on reef ecosystems, including herbivory, corallivory, bioerosion and sediment transport (Bonaldo, Hoey & Bellwood, 2014; Russ et al., 2015). The greenbeack parrotfish, *Scarus trispinosus* Valenciennes, 1840, is the largest herbivorous reef fish in the tropical and subtropical Southwestern Atlantic (SWA), occurring as a genetically homogeneous population in the continental shelf from near the Amazon River mouth southward to the rocky reefs off Santa Catarina (00°50′–28°S) (Moura, Figueiredo & Sazima, 2001; Pinheiro et al., 2018; Bezerra et al., 2018). This Brazilian-endemic parrotfish is among the most abundant reef fishes in the small and species-poor SWA coralline reefs, where it may comprise >50% of total fish biomass (Rocha & Rosa, 2001; Francini-Filho & Moura, 2008a; Francini-Filho & Moura, 2008b). Depending on body size, the greenbeack parrotfish feeds as a scraper or excavator, eating algal turfs and macroalgae (Francini-Filho et al., 2008).

In the last three decades, catch rates of large carnivorous reef fishes (e.g., snappers/Lutjanidae and groupers/Epinephelidae) declined sharply in the SWA (Fredou, Ferreira & Letourneur, 2009; Freitas et al., 2011). Up until the 1980’s parrotfishes were rarely captured by recreational fishers and were not targeted by commercial fisheries. However, in the mid 1990’s parrotfish captures intensified in Northern and Northeastern Brazil (Ferreira et al., 2012; MMA, 2014; Roos et al., 2015), with widespread usage of gillnets, traps and spears, followed by a remarkable abundance decline of *S. trispinosus* at all studied locations (e.g., Francini-Filho & Moura, 2008a; Francini-Filho & Moura, 2008b; Bender et al., 2014; Pereira et al., 2016). Its population is estimated to have declined by >50% over the past three decades, and *S. trispinosus* is currently considered one of the world’s most endangered parrotfishes (Comeros-Raynal et al., 2012).

In addition to the weak governance structures of Brazil and most other developing countries (Pomeroy & Andrew, 2011), management of large tropical reef fishes is complicated due to their gregarious behavior and spawning aggregations, longevity, slow-growth, and late-maturation (Choat, Axe & Lou, 1996; Taylor & Choat, 2014; Taylor et al., 2018). Despite such challenges, biological data may be used to implement simple management measures (e.g., size limits, seasonal closures), and may also contribute to determine productivity and resilience to exploitation (Wooton & Smith, 2014; Aschenbrenner et al., 2017; Hoffmann et al., 2017).

Here we provide novel information on age, growth and reproductive parameters of *S. trispinosus* and propose that slow-growth and longevity are major drivers of its vulnerability to over-exploitation. We determined the time and periodicity of rings’ formation in otoliths and investigated growth and size structure from catches originating at heavily fished coastal reefs and less impacted deeper offshore reefs within the Abrolhos Bank, the region where most of the remnant population of the greenbeack is concentrated (Ferreira et al., 2012). We also provide suggestions to facilitate future monitoring of age structure and to improve fisheries management based on simple indicators than can be easily acquired, understood and incorporated by stakeholders (Froese, 2004; Busilacchi et al., 2012; Aschenbrenner et al., 2017).

**Figure 1:**
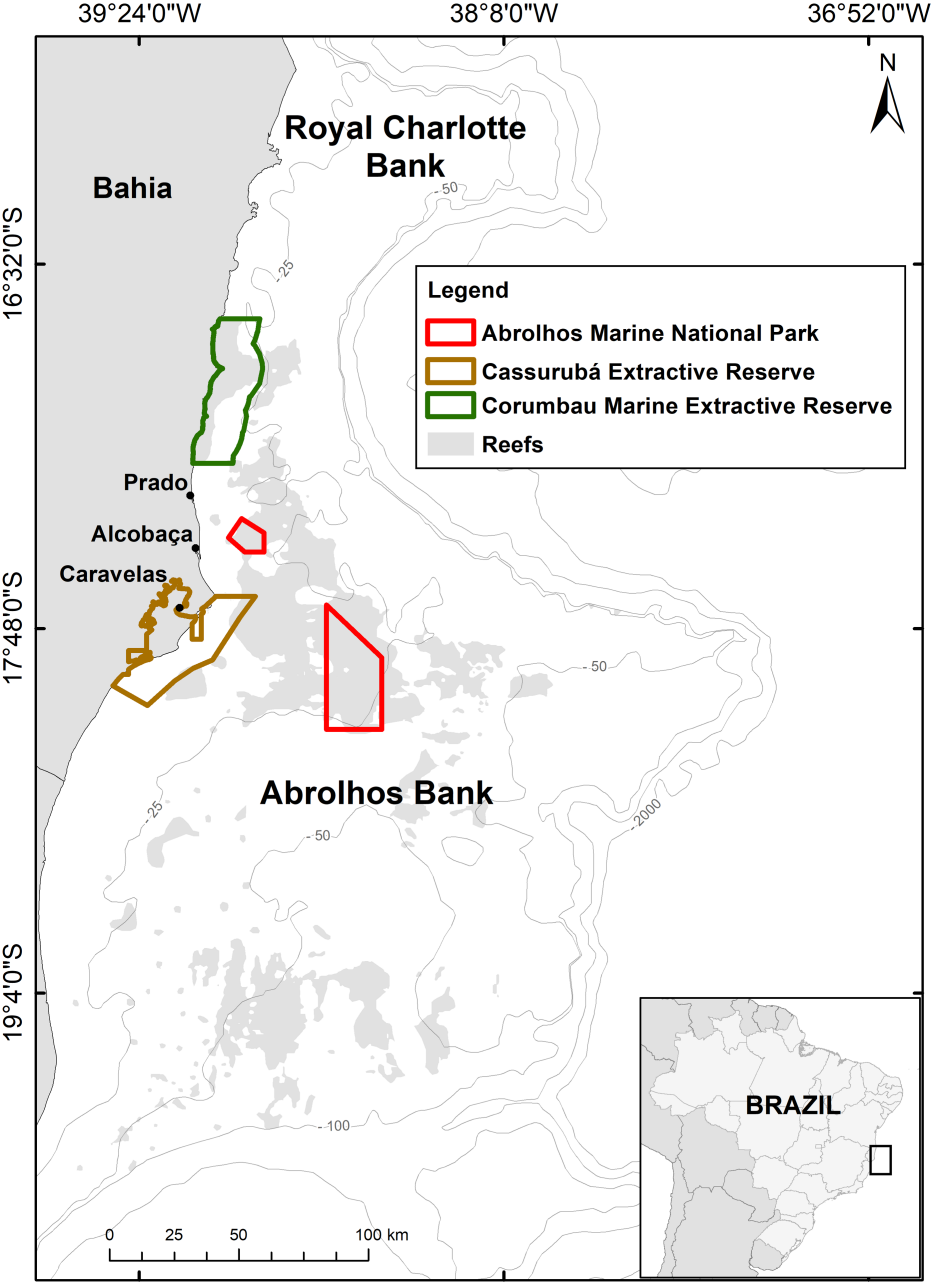
Map of the Abrolhos Bank region, Brazil. The two benthic megahabitats (reefs and rhodolith beds) and marine protected areas (MPAs) where *Scarus trispinosus* occurs are depicted, as well as the municipalities in which samples were obtained.

## Materials and Methods

### Study area

The Abrolhos Bank (16°40′–19°40′S, 39°10′–37°20′W) is a 46,000 km^2^ enlargement of the eastern Brazilian shelf (Fig. 1) with depths rarely exceeding 70 m (Moura et al., 2013). The region encompasses South Atlantic’s largest reefs (20% of the area) and rhodolith beds (∼40%), the two benthic megahabitats where parrotfishes are common. The region is under the synergistic effects of climatic and local anthropogenic stressors (Bruce et al., 2012; Ribeiro et al., 2018). Abrolhos is Brazil’s main reef fisheries ground (Freitas et al., 2014) and bears the country’s first no-take National Marine Park (910 km^2^, declared in 1983) and two community-based Extractive Reserves (ERs), Cassurubá (1,010 km^2^, 2009) and Corumbau (815 km^2^, 2000). Reef fisheries are predominantly artisanal (Freitas et al., 2011; Freitas et al., 2014), and the main landings occur in the cities of Porto Seguro, Prado, Alcobaça, Caravelas and Nova Viçosa (Fig. 1). While the fleet that lands in Porto Seguro, Alcobaça and Prado largely explore offshore deeper reefs and rhodolith beds, the fleet that lands in Caravelas and Nova Viçosa exploit mostly coastal reefs (1–20 m depth) (Freitas et al., 2018; Moura et al., 2011; Moura et al., 2013; Previero & Gasalla, 2018; Teixeira et al., 2017).

### Sampling and processing

Specimens were obtained from weekly random sampling at Alcobaça (offshore reef fisheries) and Caravelas (coastal reef fisheries) (Fig. 1), between October 2010 and September 2013. Fishes were captured with gillnets (coastal reefs) and spears (coastal and offshore reefs), and were frequently eviscerated before landing/sampling. Size-distributions (*n* = 3,106 individuals) were recorded using a randomly selected subsample encompassing >50% of the specimens at each landing event. For samples taken after evisceration (*n* = 369), TW was obtained from a linear regression between total length (TL) and total weight (TW). Individuals in the smaller size classes (13.5–30.0 cm TL, *n* = 17) were collected in Timbebas Reef (Fig. 1) in December 2012.

Gonads were fixed in 10% formalin for 24 h and stored in 70% ethanol. Gonad weight (GW) was measured to the nearest 0.01 g before dehydration in increasing alcohol concentration series, clearing in xylene, and paraffin embedding. Histological sections (4–6 µm) were stained in Harris haematoxylin and eosin. Classification of gonadal development follows Brown-Peterson et al. (2011) as: immature (never spawned), developing (developing ovaries/testis, not ready to spawn), spawning capable (developmentally able to spawn), regressing (cessation of spawning) and regenerating. The latter four stages were considered sexually mature, or adults.

Due to the absence of immature males, the TL at which 50% of individuals were mature (*L*_50_) and the minimum size at maturity (*L*_*min*_) were estimated only for females, using Bayesian methods (King, 2013; Kinas & Andrade, 2010). For *L*_50_estimates we used the logistic regression model *PMF* = 1∗(1 + *exp*(*a* + *β*∗*L*)) − 1, where *PMF* is the proportion of mature females in length class *L*, *a* is a parameter, and *L*_50_ =  − *a*∗*β* − 1. The model was implemented using uninformative priors, using the Automatic Differentiation Model Builder software (Fournier et al., 2012). Samples from the posterior distributions of parameters were obtained with a Markov Chain Monte Carlo algorithm (Gelman et al., 2004), using a 10 million chain and samples saved at every 10,000 interactions.

Reproductive cycles of sexually mature females were assessed by recording monthly changes in the gonadosomatic index (*GSI*), calculated as *GSI* = (*GW*∕(*TW* − *GW*))∗100, where GW corresponds to gonad weight. Gonadal development and *GSI* were analyzed by grouping samples from the four years of sampling in 12 months. Normality and homocedasticity of TL and *GSI* data were verified with Shapiro–Wilk and Barlett tests, and a *t*-test was used to compare TL between sexes. Sex ratios for each size class were compared with two-tailed Chi-square tests, and mean TL between sexes were compared with the Mann–Whitney U-test (Zar, 1999).

**Figure 2:**
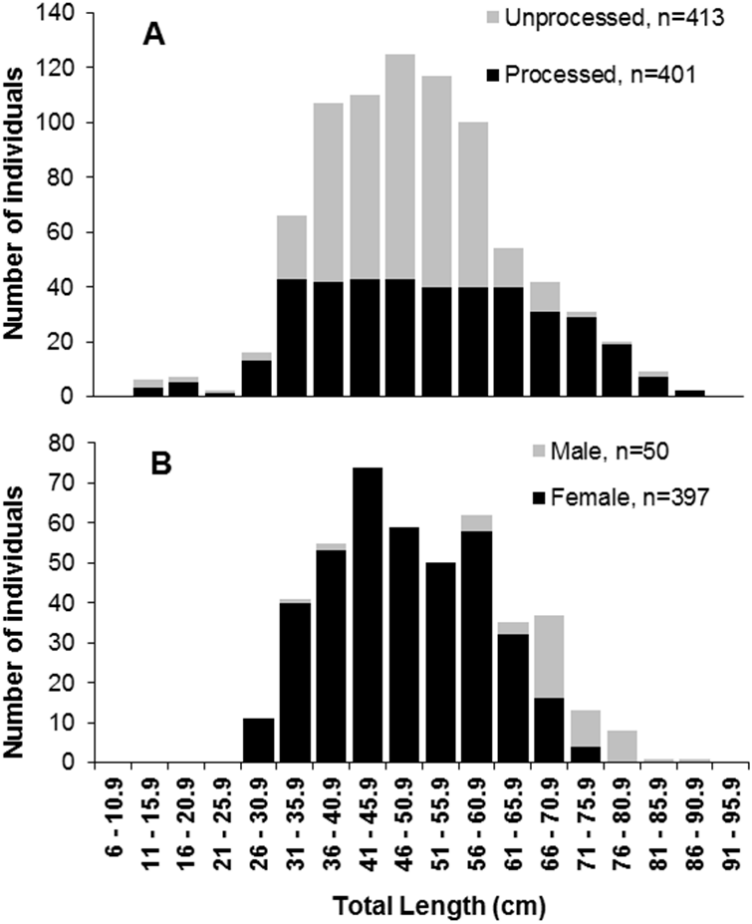
Length frequencies of *S. trispinosus* individuals captured in the Abrolhos Bank, Brazil (2010–2013). (A) Pooled samples showing the fraction of individuals whose otoliths were removed and processed. (B) Samples discriminated by sex. Significance level of 5% (*χ*2 > 3,841) Chi^2^ *two tailed*; * ratio with zero.

A subsample of 401 individuals (13.5–86.0 cm TL) (Fig. 2A) with similar numbers of individuals at each five cm size class was selected for assessing age and growth parameters (Lai et al., 1996). Right side sagittae otoliths were removed, washed and dried, weighted to the nearest 0.1 g and embedded in polyester resin UN 1866 (Secor, Dean & Laban, 1991; Panfili, Troadec & Wright, 2002). Sectioning (0.40 mm) was performed with a Buehler-Isomet metallographic saw. Cuts were performed transversely through the core, from the inner to the outer face of the otolith, and were subsequently fixed on glass slides (Secor, Dean & Laban, 1991). Sections were photographed with a Digital Microscope Camera OPT5000 and images were processed following Campana (2001). The number and distance between rings were obtained along an axis from the core to the dorsal region of the otolith (light bands considered age rings), with two independent readings in random order and without prior knowledge about the specimen (Campana, 2001).

Accuracy of readings was assessed by calculating the average percentage error (APE), following Beamish & Fournier (1981) and Campana (2001). Trends between readers were determined by calculating the probability of disagreement (PD) and average magnitude of inter-reader discrepancies (IRD) (Marriott & Mapstone, 2006). A marginal ratio increase (MRI) analysis was performed to assess ring formation periodicity, using individuals <50.0 cm TL (last rings of older animals are closely spaced and difficult to read). The MRI analysis assumes no significant differences in otolith growth between adjacent rings, and consists in the computation of the ratio between the growth after the formation of the last ring, and between the second to the last ring (Campana, 2001; Williams, Davies & Mapstone, 2005). Differences in marginal increases between two subsequent seasons were assessed with a *t*-test (Campana, 2001). Growth was modeled with the von Bertalanffy growth function (VBGF) fitted by minimizing the sum of squares using the Gauss-Newton algorithm (Beverton & Holt, 1957), with the length *L* of an individual *j* with age *t* (*L*_*t*,*j*_) corresponding to *Lt* + ε_*t*,*j*_, where ε_*t*,*j*_ is a random variable that follows a normal distribution with mean equaling zero and variance *σ*^2^. The VBGF parameters were estimated for the first and second readings, for the average of the readings, for males and females pooled, and for each sex separately. Curves for both sexes were compared using Kimura’s likelihood ratio test (Haddon, 2001). Differences in growth of fish from different cross shelf strata (offshore and inshore) were investigated using back-calculated TL and ages obtained in the second reading, using the Allometric Biological Intercept (ABI) model (Vigliola & Meekan, 2009). This model assumes proportional growth rates of otolith and fish, and is calculated using otolith radius and the distance between the core and each age ring. The relationship between otolith weight and age was calculated and compared with those of other scarins from which growth ring periodicity was validated by tetracycline labeling (Choat, Axe & Lou, 1996; Taylor & Choat, 2014).

The minimum age at which females mature (*A*_*min*_) was recorded, and the age at which 50% of females were mature (*A*_50_) was estimated by fitting the logistic regression model *P* = 100∕(1 + *exp* − *r*∗(*A* − *A*_50_)), where *P* is the percentage of mature fish on age-class *A* and *r* is the maturity width (King, 2013). To predict the probability that an individual is mature based on its length, observations (0 = immature, 1 = mature) and TL were fitted to a binary logistic model, with further construction of maturity ogives (maturity-at-length probability plots). Generation time (*GT*) was estimated with the equation *GT* = *A*_50_ + (*Z*∗(*Longevity* − *A*_50_)), where *Z* is the total mortality rate and *A*_50_ is the mean age at which 50% of the population is mature. Calculations were performed using the Fisheries Stock Assessment (FSA) package (Ogle, Wheeler & Dinno, 2018) in R 3.0.1 (R Development Core Team, 2008). Natural mortality (*M*) was estimated for each cross-shelf strata from equation ln(*M*) = *a* + *b*ln(*t*_*max*_), where *t*_*max*_ is the maximum TL in the sample (Hoenig, 1983). Fishing mortality (*F*) was obtained from the difference between total mortality (*Z*) and *M*. For comparative purposes, *M*, *Z* and survival (*S*) were also calculated following Chapman & Robson (1960), Alverson & Carney (1975) and Pauly (1980). The status of the studied stock was assessed with three simple indicators (Froese, 2004), all of them based on length distribution and/or optimum length (*L*_*opt*_), as follows: (i) percentage of mature specimens (>*L*_50_) in the catch; (ii) percentage of fish caught at ±10% of the optimum length (*L*_*opt*_), obtained empirically with the equation *L*_*opt*_ = 3∗*L*_∞_∕3 + *M*∕*k* (Beverton, 1992); (iii) percentage of large fish in the catch (>1.1 *L*_*opt*_).

Collections were made under SISBIO permit #45738-2.

**Figure 3:**
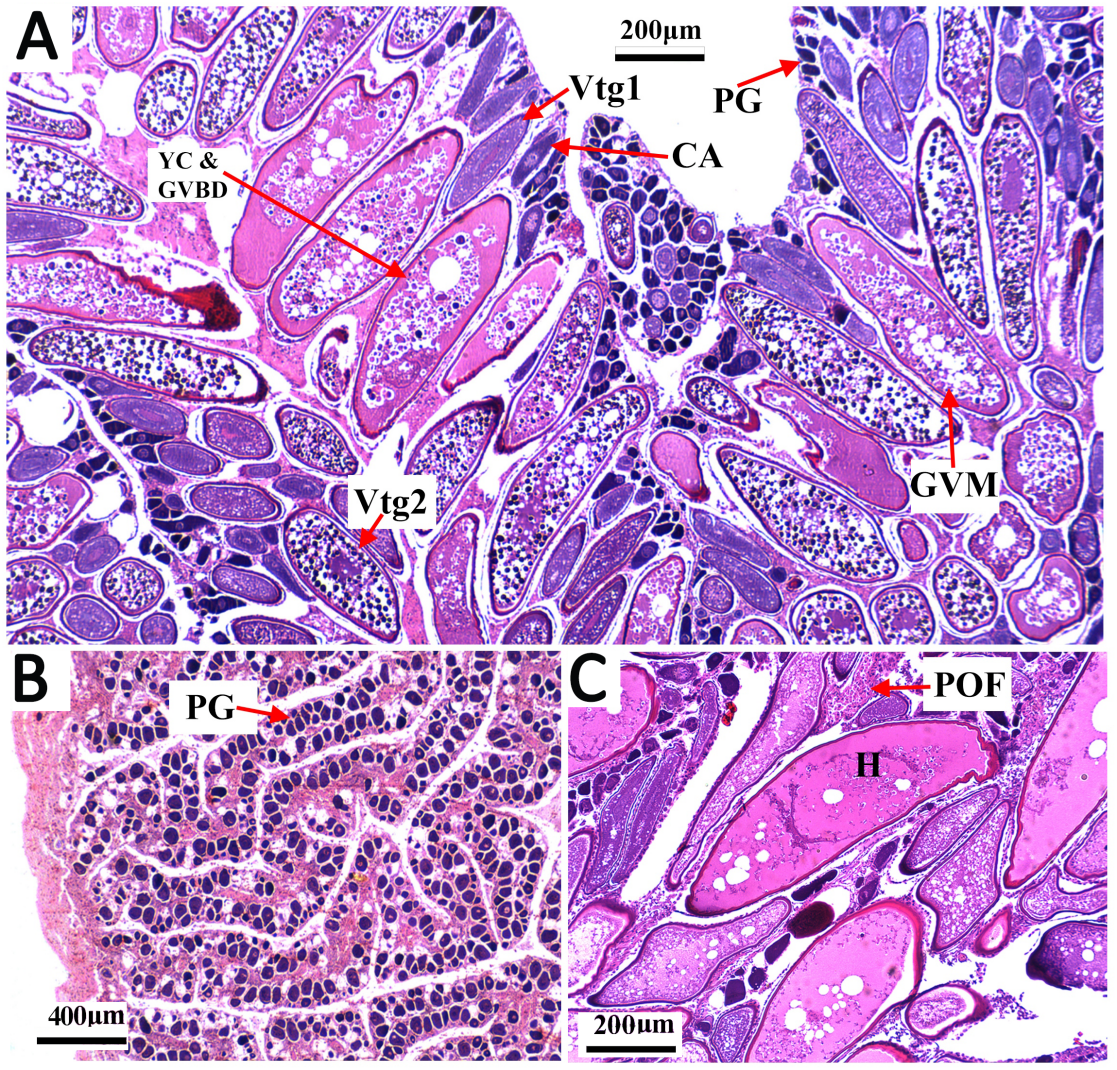
Transverse thin sections of *S. trispinosus* ovaries. (A) Spawning capable ovary with oocytes under different development stages: PG (primary growth), CA (cortical alveolar), Vtg1 (primary vitellogenesis), Vtg2 (secondary vitellogenesis), GVM (germinal vesicle migration), and YC & GVBD (yolk coalescence and germinal vesicle breakdown). (B) Regenerating ovary. (C) Detail of a hydrated oocyte (H) and a Postovulatory Follicle (POF).

## Results

### Reproductive biology

Most gonads (89%) were ovariform (*n* = 405), while the remaining (11%) were testiform (*n* = 50). This overall 1:8 (male:female) sex ratio became biased toward males in the largest size classes (66.0–86.0 cm TL) (*χ*^2^, *p* > 0.05) (Fig. 2B). Total Length (TL) and Weight (TW) ranged between 33.0–86.0 cm and 1,000–11,111 g for males, and 26–75 cm and 118–7,355 g for females. Males’ mean TL (69.0 ± 9 cm) and TW (5,432 ± 1,708 g) were significantly higher than those of females (TL 47.0 ± 10 cm; TW 2,068 ± 1,506 g) (*t*-test, *p* < 0.05).

**Figure 4:**
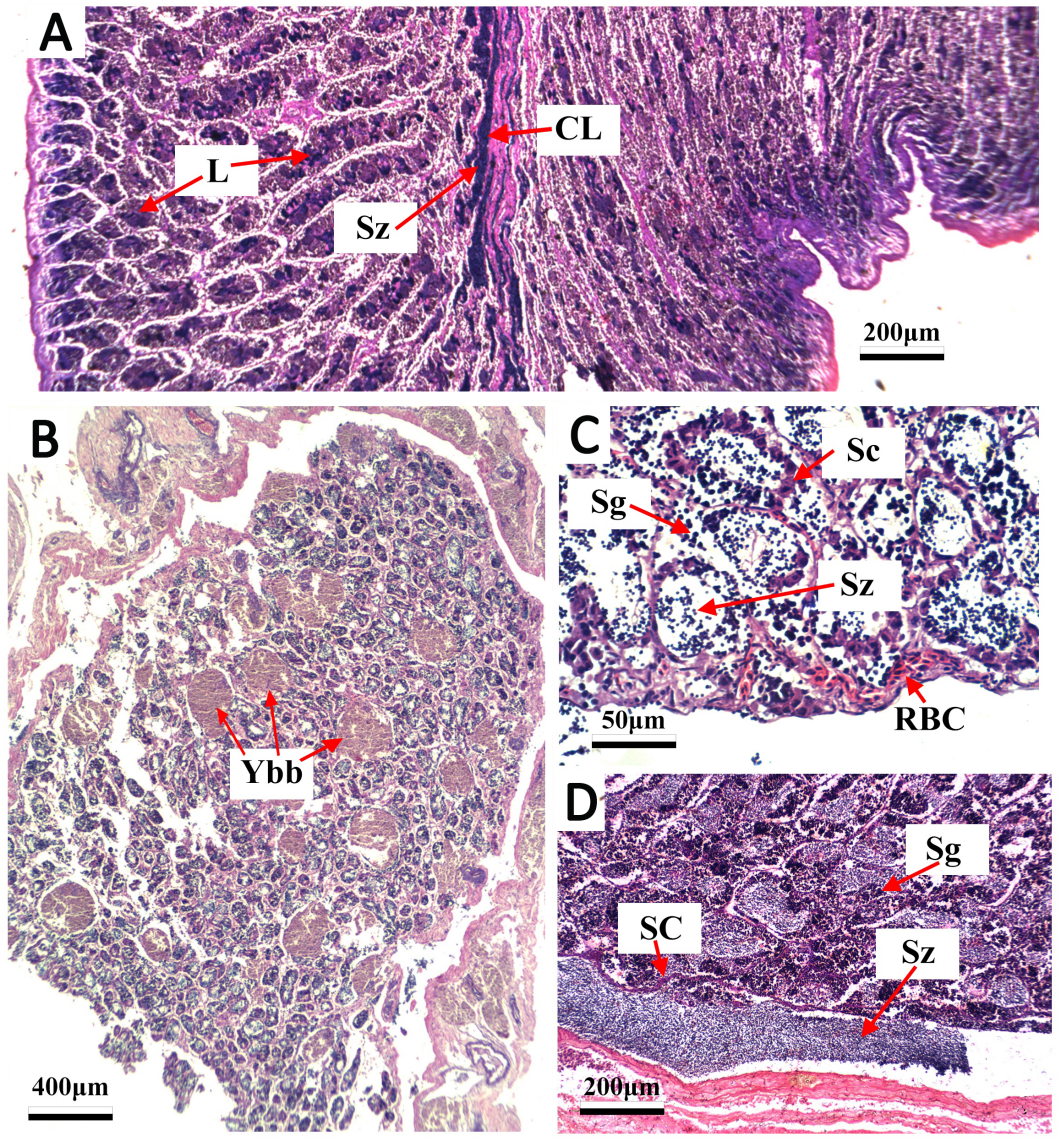
Transverse thin sections of *S. trispinosus* testicles. (A) Spawning capable with central lumen (CL) full of spermatozoa (Sz) and lumen of lobules (L); (B) developing phase with few Sz and several yellow-brown bodies interspersed in the lamellas; (C) developing phase with Sz, Spermatogonia (Sg), sertoil cels (Sc) and red blood cells (RBC).

Gonads were bilobed and caudally united with the ova/sperm duct. Oocytes presented a conspicuous elliptical shape in all stages. Groups of oocytes under different developmental stages were detected in all adult females, indicating asynchronous oogenesis. Immature females had small ovaries with ovigerous lamellae filled with primary growth oocytes and narrow lumens. Developing females had bigger and more vascularized ovaries with different stages of early development oocytes (cortical alveolar, primary and secondary vitellogenic) (Fig. 3A). Spawning capable females had large and well-vascularized ovaries with oocytes ranging from early to advanced developmental stages (tertiary vitellogenic and hydrated oocytes, yolk coalescence, germinal vesicle breakdown, postovulatory follicles) (Fig. 3C). Regenerating females had small gonads with empty lumens and spaces between oocytes in lamellas, presenting only primary vittelogenic and cortical alveolar oocytes (Fig. 3B). Several spawning capable and regenerating individuals presented granular yellow-brown bodies among oocytes, with similar shapes and sizes. Spawning capable males had large and firm testes with lobular lumens and sperm ducts full of spermatozoa (Fig. 4A). Regenerating males had threadlike and reduced testes with few spermatogonia and vestiges of sperm in the central duct, which lacked lobules. Testes had seminiferous tubules arranged in lamellas and a well evident central sperm duct (Fig. 4). Developing males presented spermatogonias and few spermatozoa in the lumen of seminiferous tubules. Some testes also presented yellow-brown bodies interspersed in the lamellas (Figs. 4B and 4C).

**Figure 5:**
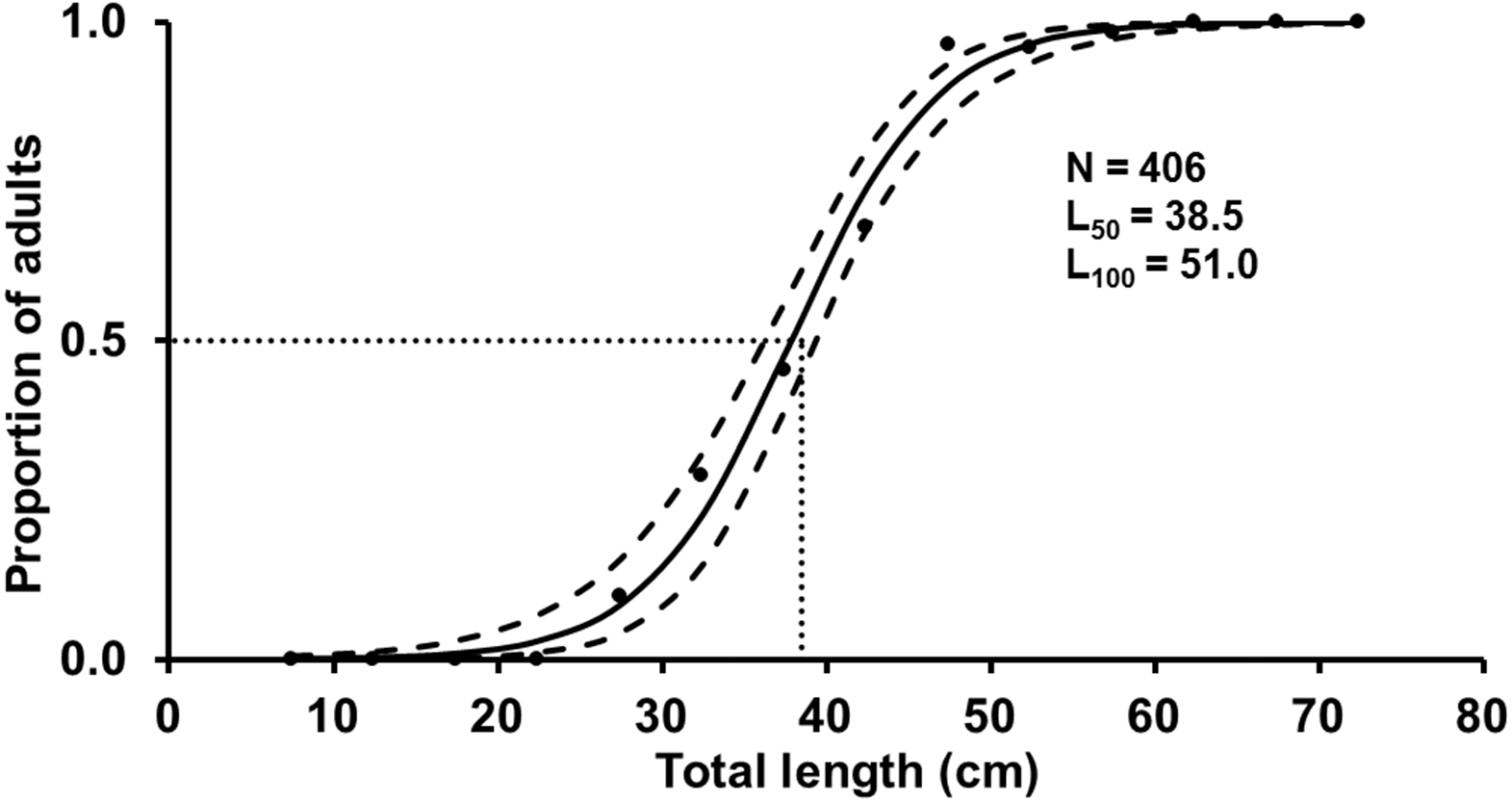
Estimated logistic regression of the proportion of mature *S. trispinosus* females from the Abrolhos Bank, Brazil (maximum of the posterior distribution). Black lines indicate the length at which 50% of the individuals were mature (mean of the *L*_50_ posterior distribution) and grey lines indicate the 90% of credibility interval. Black dots represent the observed proportion of mature females.

**Figure 6:**
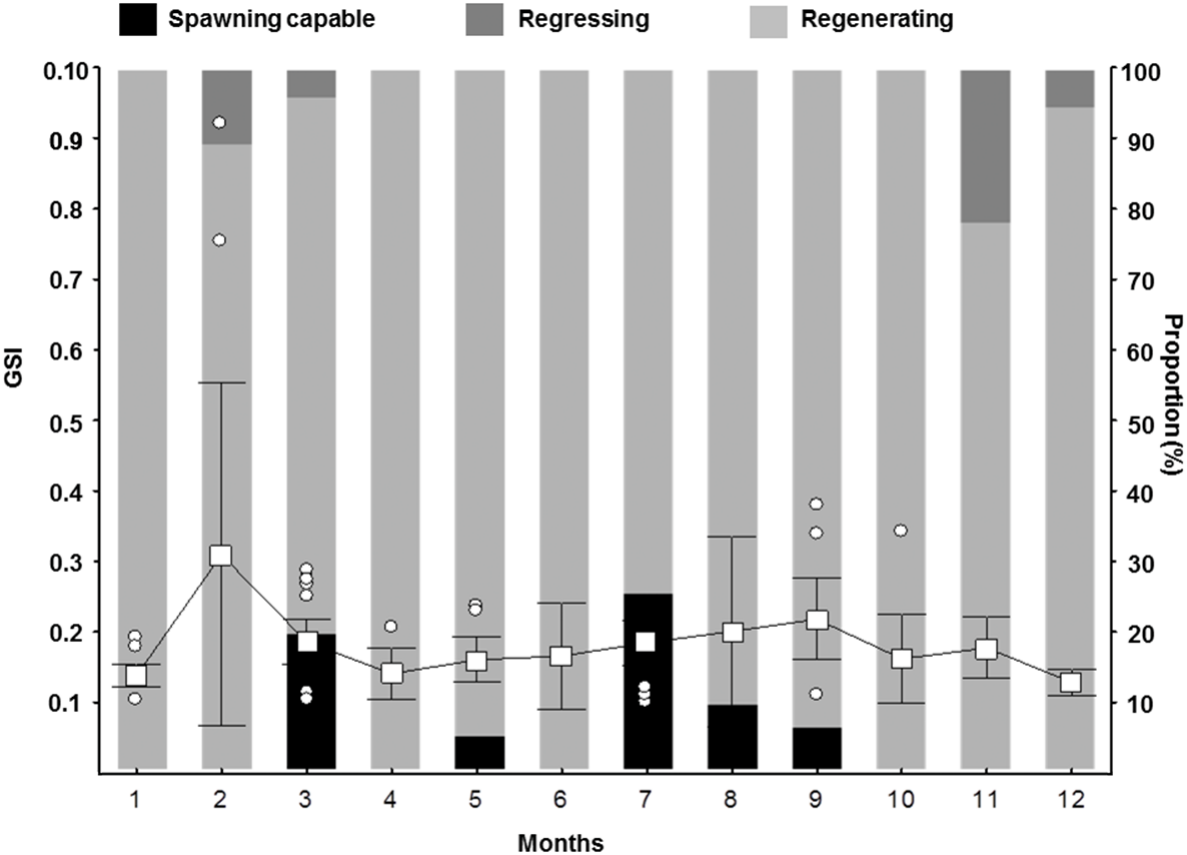
Gonadosomatic Index (GSI) for female *S. trispinosus* captured in the Abrolhos Bank, Brazil (2010–2013). Outliers are shown as circles, means as squares, and bars represent standard deviation (0.95%).

The *L*_100_ for females was 51.0 cm TL and the estimated *L*_50_ was 38.5 cm TL (mean of the posterior distribution), with the 90% CI between 37.4–39. one cm TL (Fig. 5). Spawning capable males and females were present year-round, and variation in GSI (females) indicates two reproductive peaks: February-March, and June-September (Fig. 6).

### Age and growth

The first otoliths’ readings recorded 2–22 rings, and the second 1–22 rings. Most otoliths (81.2%) had 4–9 rings. Specimens >70 cm TL generally had >8 rings (Supplemental Information 1). The APE between readings was 8.33% (CV = 11.78%), with higher PD in older classes. The IRD ranged between 0.5–1 for most ages, reaching 3 for readings of older individuals. The MRI analysis indicated growth marks formed annually between November-December, with significant differences in marginal increment between November-January (t test for independent samples = 5.39, *p* = 0.03) (Supplemental Information 2).

**Figure 7:**
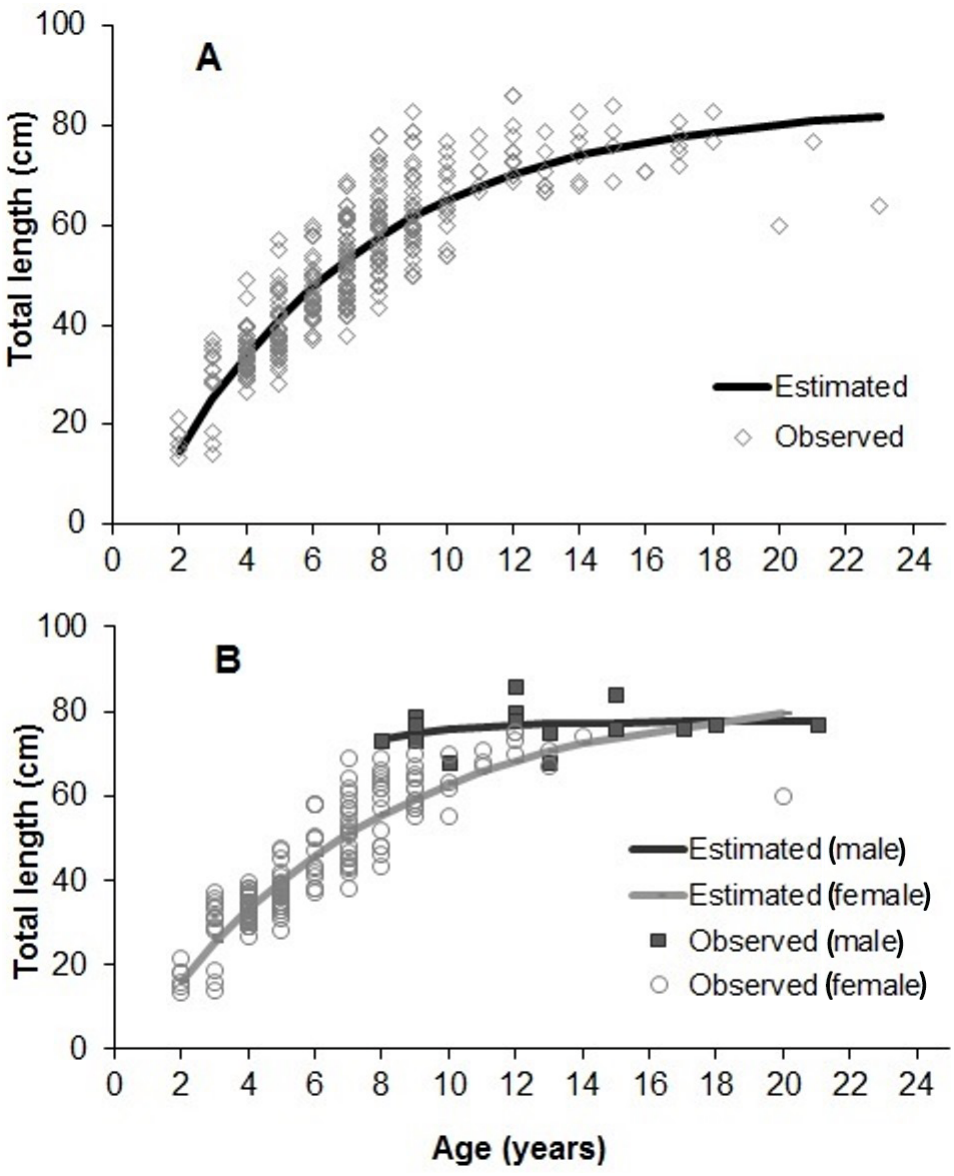
Von Bertalanffy growth estimated from 358 individuals of *S. trispinosus* from the Abrolhos Bank (adjusted for readings 1 and 2). (A) All samples pooled. (B) Separate estimates for males and females.

The adjustment of growth parameters to observed data is presented in Fig. 7. Growth parameters (sexes pooled) were 85.28 cm (*L*_∞_), 0.14 years^−1^ (K) and 0.16 years (t0) (Tables 1 and 2). Parameters estimated for each sex and area (inshore/offshore) showed significant lower *L*_∞_ and K for females, and also for specimens from inshore reefs (Tables 1 and 2, Fig. 7).

**Table 1:** Parameters of von Bertalanffy growth (VBG) estimates for the *Scarus trispinosus* specimens sampled in the Abrolhos Bank, Brazil (second otoliths’ readings). Each line indicates the growth parameters for each sex and cross-shelf strata (inshore and offshore).

*Sex/area*	*L*_∞_ (cm)	S.E.	*K* (year^−1^)	S.E.	*t*_0_ (year)	S.E.	S.D. of residuals	Total mortality (Z)
Grouped sexes	85.28	4.59	0.14	0.02	0.16	0.40	10.30	
Male	80.10	4.59	0.25	0.09	0.54	1.21	9.65	
Female	77.46	8.05	0.17	0.05	0.40	0.68	9.57	
Inshore reefs	42.48	2.05	0.44	0.10	0.56	0.35	5.97	0.76
Offshore reefs	85.75	7.10	0.11	0.03	−2.62	1.70	8.96	0.25

**Table 2:** Kimura test by sex, area and grouped sexes for second otolith’s readings of *Scarus trispinosus* from the Abrolhos Bank, Brazil. Lines describe the alternative hypothesis of differential growth among sites and sexes.

Males and Females				
Tests	Hypothesis	Chisq	*df*	*P*
1 Ho vs H1	Linf1 = Linf2	3.31	1	0.069
2 Ho vs H2	K1 = K2	0.05	1	0.823
3 Ho vs H3	t01 = t02	0.54	1	0.462
4 Ho vs H4	Linf1 = Linf2, K1 = K2, t01 = t02	15.84	3	0.001

Tests	Hypothesis	Chisq	*df*	*P*
1 Ho vs H1	Linf1 = Linf2	3.98	1	0.046
2 Ho vs H2	K1 = K2	1.81	1	0.179
3 Ho vs H3	t01 = t02	3.11	1	0.078
4 Ho vs H4	Linf1 = Linf2, K1 = K2, t01 = t02	44.93	3	0.000

**Figure 8:**
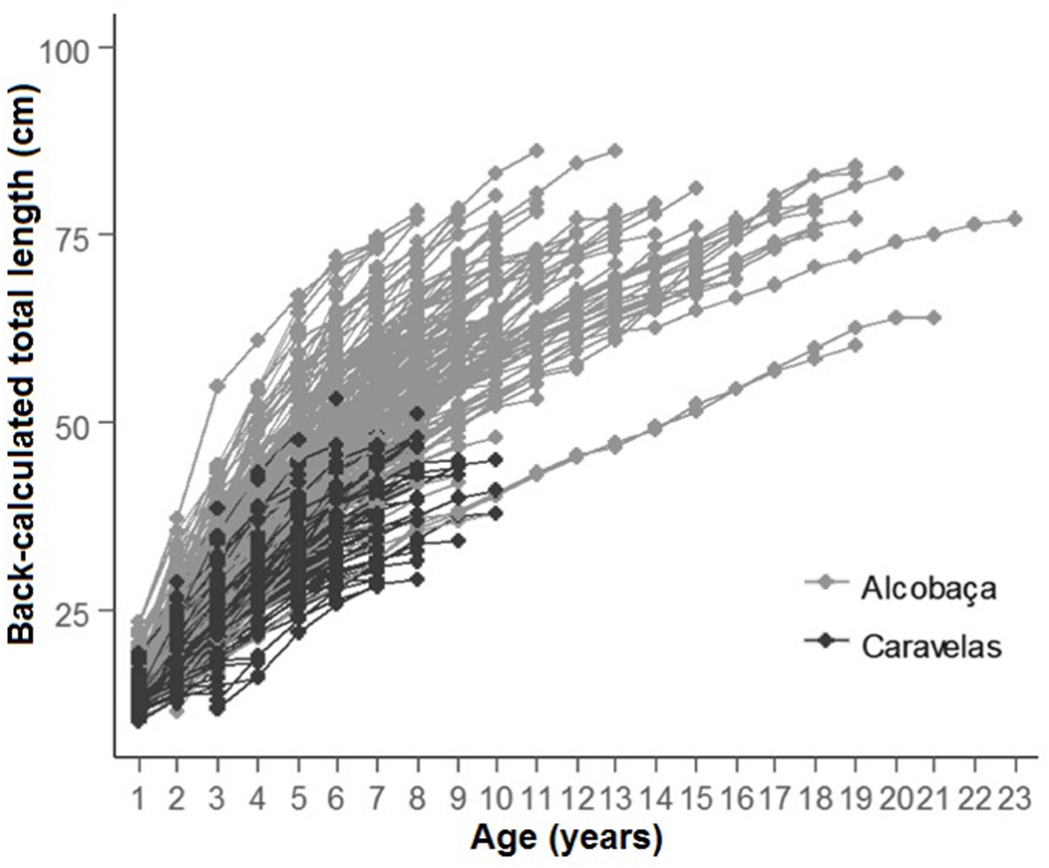
Back-calculated lengths at age for *S. trispinosus* individuals from Alcobaça (offshore reefs) and Caravelas (inshore reefs). Ages are based on the second otolith readings.

Growth was remarkably fast in the initial 20% of the life-span, when 50% of *L*_∞_ was achieved. Retro-calculated ages of specimens from inshore and offshore reefs/rhodolith beds evidenced faster-growing and older individuals in the latter (Fig. 8). Only individuals <9 years were captured in inshore reefs, while most individuals with 3–22 years came from offshore reefs/rhodolith beds (Fig. 8). The relationship between otolith weight and age was linear and similar to those of other parrotfishes for which annual ring formation has been validated (Supplemental Information 3).

**Figure 9:**
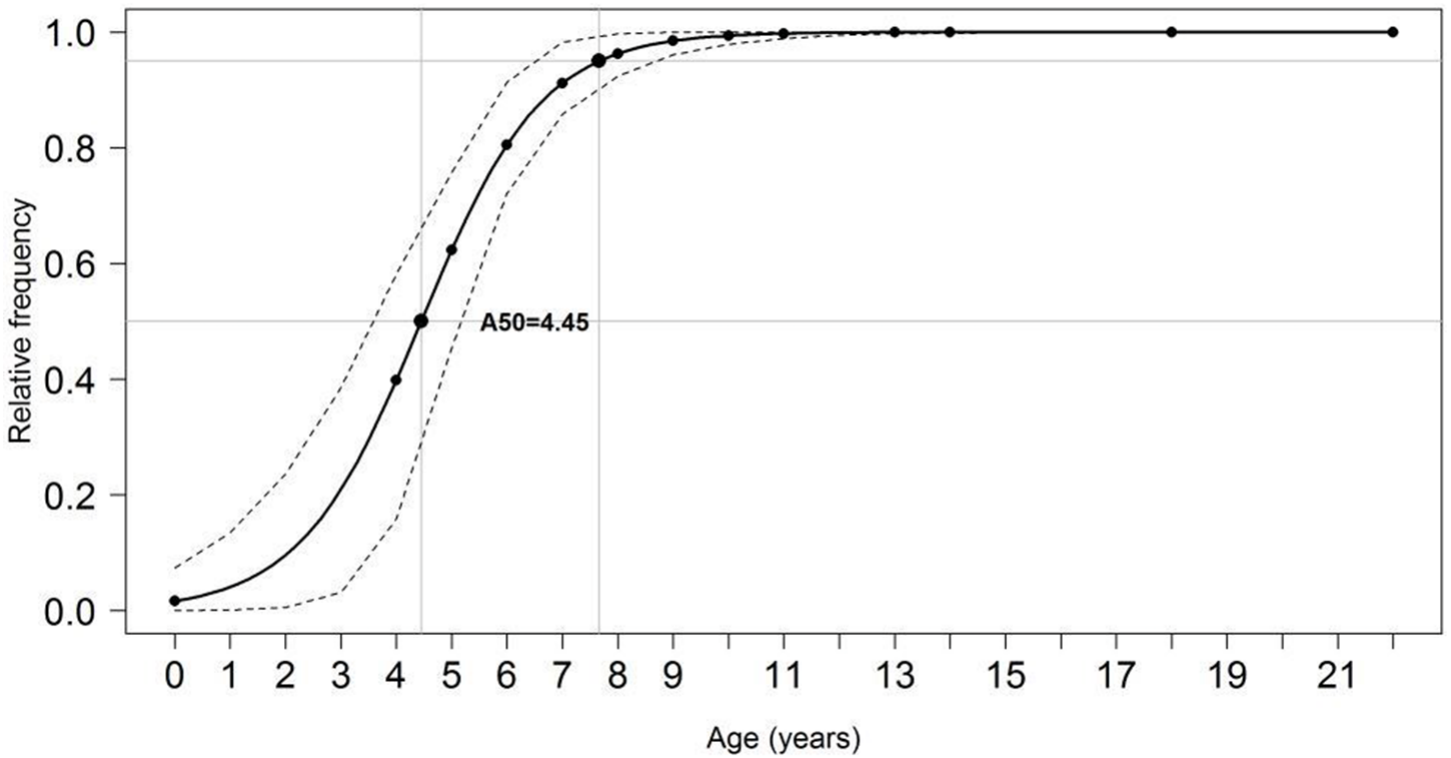
Proportion of mature individuals of *S*. *trispinosus* at each age. Dashed lines indicate age at which 50% (*A*_50_) and 95% (*A*_100_) of individuals were mature.

For pooled samples (males and females, inshore and offshore), *A*_50_ and *A*_100_were estimated at 4.45 and 11 years (95% CI), respectively (Fig. 9). Total mortality (Z) ranged between 0.87–0.92, natural mortality (M) between 0.17–0.35 (22% of Z), survival *(S)* between 0.40–0.42, and fishing mortality (F) was estimated at 0.68 (78% of Z, or 3.5 times larger than *M*). Estimated generation time was 20.6 years.

**Figure 10:**
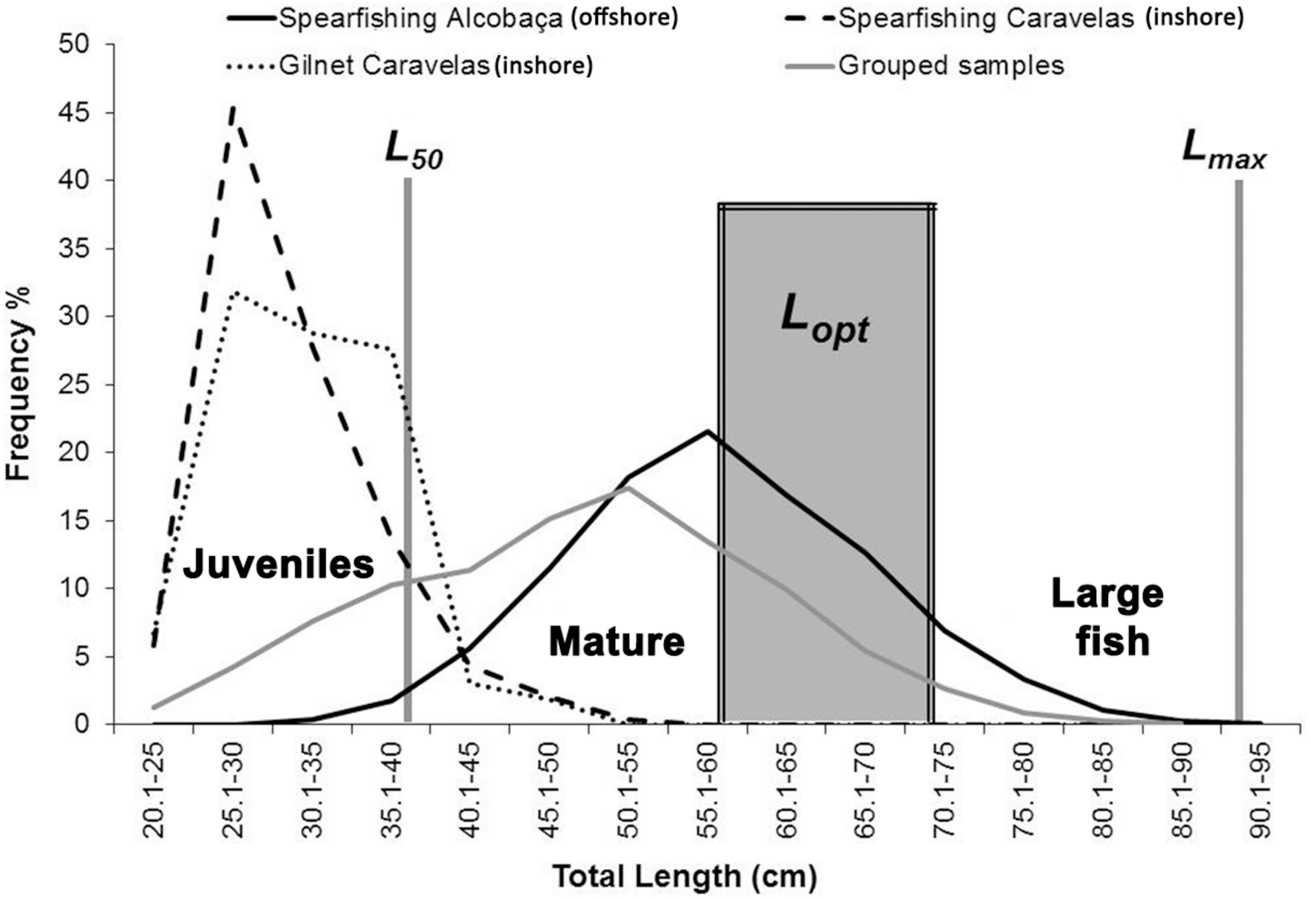
Length–frequency for *S. trispinosus* landings (2011–2013) in the Abrolhos Bank, Brazil. Data is presented separated by fishing type and locality (inshore and offshore fisheries). *L*_*m*_ indicates length at first maturity, *L*_*opt*_ indicates the length range where maximum yield could be obtained and *L*_*max*_ is the maximum size (*n* = 3,106).

Juvenile, adult, *L*_*opt*_ and large fish (>1.1 *L*_*opt*_) specimens corresponded to 10.6, 89.4, 28.4 and 7.0% of all landings (inshore and offshore), respectively (Fig. 10). Samples obtained by spearfishing in offshore reefs included 1.1% of juveniles and 98.9% of adults (*n* = 2,458). Large fish (>1.1 *L*_*opt*_) and *L*_*opt*_ specimens captured with spearfishing in offshore reefs and rhodolith beds represented 8.9 and 35.8% of adults, respectively. In samples obtained by spearfishing in inshore reefs, juveniles corresponded to 41.6%, while adults represented 58.4% (*n* = 485). Large fish (>1.1 *L*_*opt*_) were absent from inshore reefs, and *L*_*opt*_ individuals represented only 1.6% of the landings. In gillnet fisheries, performed only inshore, juveniles and adults corresponded to 61.3 and 38.7% of the landings, respectively (*n* = 163). Specimens >*L*_*opt*_ were absent.

## Discussion

**Figure 11:**
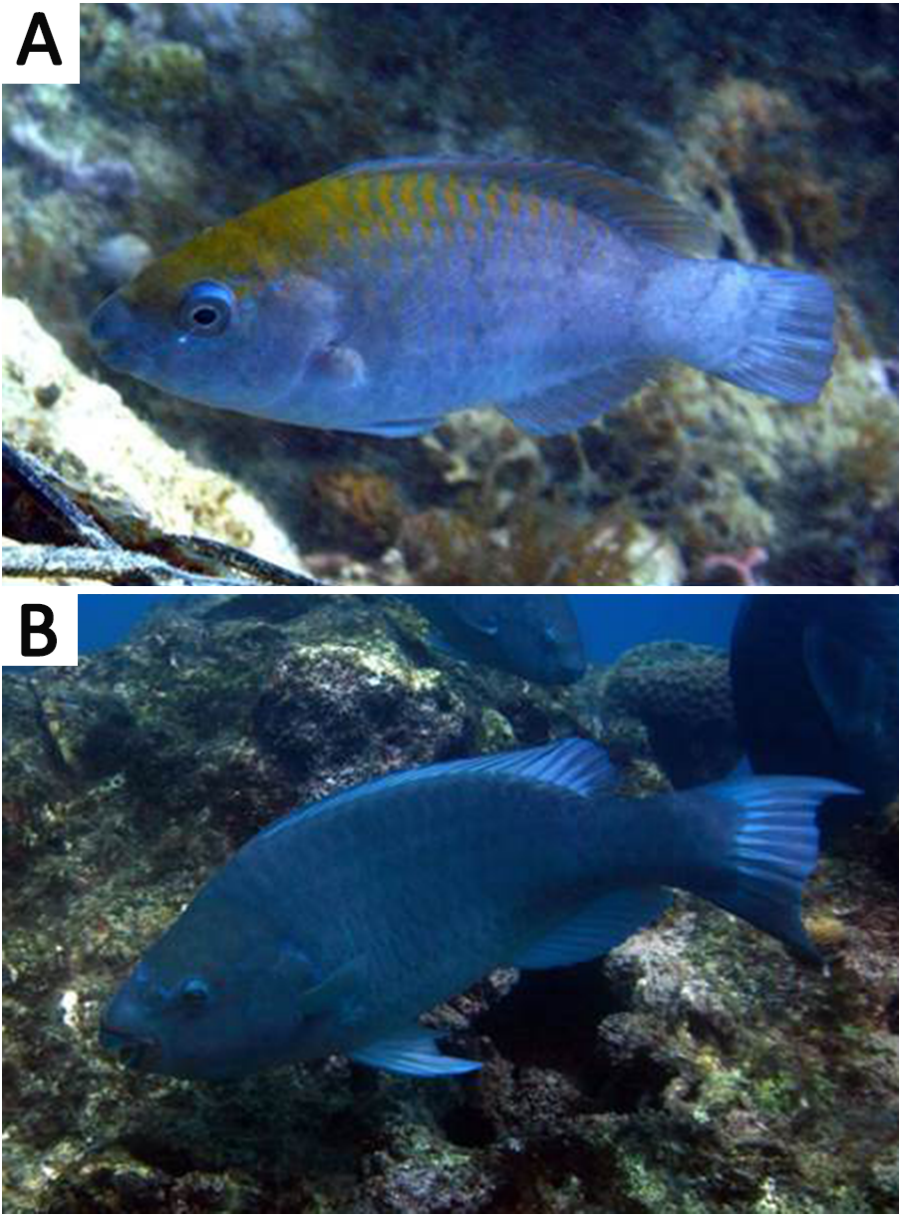
Color pattern of *Scarus trispinosus*. (A) Juvenile specimen showing yellow area on nape. (B) Adult individual. Both images from the Abrolhos Bank reefs, Brazil. Photo by Ronaldo B. Francini-Filho.

The histo-morphological aspect of testicles, presence of yellow-brown bodies in Developing testicles, sex ratio strongly biased toward females, and only males comprising the largest size classes indicate that *S. trispinosus* is a protogynous hermaphrodite. We did not record external sexual dimorphism enabling discrimination of IP and TP individuals, but the presence of small and potentially primary males (<*L*_50_) indicates that *S. trispinosus* may be diandric (Fig. 2B), a condition that is often associated with the presence of territorial TP males that access females by interference (Warner, 1975; Robertson & Warner, 1978; Robertson, Reinboth & Bruce, 1982; Streelman et al., 2002). However, territoriality in *S. trispinosus* seems to be weaker, or at least less frequent, than that displayed by other diandric labrids (Buckman & Ogden, 1973; Van Rooij, Kroon & Videler, 1996; Mumby & Wabnitz, 2002). The overall sex ratio of *S. trispinosus*, biased toward females (1:8), is typical of other protogynous hermaphrodite reef fishes, either monandric or diandric (HCFRU, 2008; Ebisawa et al., 2016), but protogyny needs confirmation (Sadovy-de Mitcheson & Liu, 2008). Similarly to its two closely related species, *S. guacamaia* and *S. coelestinus,* color pattern of juvenile *S. trispinosus* resembles that of adults, except for the presence of inconspicuous whitish stripes and a yellowish area on nape in small juveniles (<5.0 cm TL) (Fig. 11). Together with the consistent co-occurrence of different reproductive stages, the temporal variation in the GSI indicates that the reproduction of *S. trispinosus* occurs year-round, with increased activity in February-March and August-September (Fig. 6). This pattern agrees with the reproductive seasonality of most parrotfishes, with increased spring-summer activity (HCFRU, 2008; Afonso, Morato & Santos, 2008; Ebisawa et al., 2016). When compared to other large roving herbivorous reef fishes, such as surgeonfishes (Acanthuridae), which reach >40 years, parrotfishes are shorter-lived, with life spans between 5–30 years (Choat, Axe & Lou, 1996; Taylor & Choat, 2014; Taylor et al., 2018). *Scarus trispinosus* is not only one of the world’s largest parrotfishes, but is also among the most longevous, reaching >20 years) (Choat et al., 2006; Taylor et al., 2018).

Otolith banding can be hard to detect in tropical fishes (Fowler et al., 1995; Green et al., 2009), but there is no evidence for increment formation that is not annual among parrotfishes (Choat, Axe & Lou, 1996; Choat & Robertson, 2002; Taylor & Choat, 2014). Here, we used evidence from marginal increments along the year to show that *S. trispinosus*’ bands form annually between November and December. This timing coincides with its main reproductive peak (Fig. 6), when energetic trade-offs between growth and gamete production are expected to be stronger (Sadovy, 1996; Wooton & Smith, 2014). The average percentage error (APE) between otolith readings (8.33%, CV = 11.78%) remained within the intermediate degree of accuracy (Campana, 2001) and the magnitude of inter-reader discrepancies (IRD) (0.5–1.5 for most ages) was also relatively low, reaching only 3 for individuals >20 years. Akin to other parrotfishes, *S. trispinous* presented a linear relationship between otolith weight and age, which was less variable than the range of sizes at age (Supplemental Information 3).

Our VBGF presented a good fit (Fig. 7), but TL variation within some age classes (e.g., 5–9 years) was high, tending to stabilize around 70 cm from 9–10 years onwards. For instance, the older individual (22 years) attained 77 cm TL, while the two largest individuals (86 cm TL) had intermediate ages (10 and 12 years). This pattern is frequent among fishes (e.g., Sainsbury, 1980; Pauly, 1990), and can be related to either spatial differences in growth rates or to the presence of multiple cohorts. Co-occurrence of different cohorts is expected in *S. trispinosus*, as it spawns year-round, and large sized individuals may perform regional level movements from deeper to shallower areas (Francini-Filho & Moura, 2008a). Spatial variation in growth and other demographic patterns among parrotfishes has been associated to sex, fishing effort and resource quality/availability (Gust, Choat & Ackerman, 2002; Taylor & Choat, 2014; Lessa et al., 2016). Across the Abrolhos Bank, there is high cross-shelf variability in environmental conditions and fishing effort (Bruce et al., 2012; Freitas et al., 2011; Freitas et al., 2014; Moura et al., 2011; Moura et al., 2013; Ribeiro et al., 2018), coinciding with the sharp demographic partition between our samples from coastal (slower-growth and ages <9 years) and offshore reefs (faster growth and ages between 3–22 years) (see Fig. 8 and Table 1).

Juvenile *S. trispinosus* are absent from estuaries (Moura et al., 2011; Giglio & Freitas, 2013), but occur from coastal reefs and nearshore tidepools to offshore reefs and rhodolith beds (>20 m depth). Conversely, its sister species *S. guacamaia* is frequent (as juveniles) on mangroves and estuaries, and the closely related *S. coelestinus* (5–75 m depth range) is more abundant along inshore reef flats than in deeper areas (Mumby et al., 2004; Dorenbosch et al., 2006). Regional-scale movements of *S. trispinosus* seem to be performed only by adults (Francini-Filho & Moura, 2008b), and may be driven by density-depend and/or reproductive processes. The demographic structuring recorded herein (see Fig. 8 and Table 1) may be associated to differential food quality in inshore and offshore reefs, as well as in rhodolith beds. *Scarus trispinosus* feeds preferably on turf algae and crustose coralline algae (CCA) (Francini-Filho et al., 2010), but inshore reefs and rhodolith beds are largely covered by a frondose macroalgae canopy, especially during summer (Amado-Filho et al., 2012; Brasileiro et al., 2016). In the late winter, macroalgae are partially washed from these habitats, which may become more suitable for feeding on turf and CCA, and therefore elicit feeding and reproductive movements during spring and early summer.

Distance offshore is a good overall predictor of fishing effort (Teixeira et al., 2017), and the Abrolhos’ coastal reefs are indeed subjected to longer-term and heavier fishing effort than offshore reefs (Previero & Gasalla, 2018). The region’s fisheries are among the oldest export-oriented economic activities in Brazil, with salted groupers being the most important product of the Porto Seguro Province in the 16th Century (Bueno, 1999). However, up until recently reef fisheries have been concentrated in carnivores captured with hook-and-line or longlines in nearshore reefs, which are easily located for being visible from the sea surface. Herbivorous fish captures followed the introduction of snorkeling and spearfishing by tourists in the late 1980’s (Ferreira & Gonçalves, 1999), and offshore reef fisheries are associated with the popularization of GPS and illegal usage of SCUBA and diving hookahs from the late 1990’s on. Indeed, the deeper non-emerging reefs and rhodolith beds of Abrolhos, which represent >80% of the region’s reef fishing grounds, were not mapped until recently (Moura et al., 2013). Therefore, the cross-shelf demographic partition detected herein seems to be related to both fishing effort/gear and habitat quality/adult’s movements. The Alcobaça landings (larger and faster-growing fishes) came largely from spearfishing in offshore reefs, while the Caravelas landings (smaller and slower-growing fishes) came from gillnetting and spearfishing in coastal reefs. The “Rosa Lee phenomenon” caused by fishing, which first selects the largest individuals (Lee, 1920), may also account for the slower growth of older individuals, which do not correspond to the largest individuals in our sample (Ricker, 1975; Sinclair, Swain & Hanson, 2002).

**Table 3:** Data-poor indicators (Froese, 2004) for the different area and fisheries for *Scarus trispinosus* in the Abrolhos Bank, Brazil.

**Indicator**	**Pooled samples**	**Coastal reefsspearfishing**	**Coastal reefsgillnet**	**Offshore reefs**	
I = percentage of mature specimens (>*L*_50_) in the catch	10.6%	41.6%	61.3%	1.1%	
II - percentage of fish caught at ±10% of the optimum length (*L*_*opt*_)	28.4%	1.6%	0%	35.8%	
III = percentage of large fish in the catch (>1.1 *L*_*opt*_)	7.0%	0%	0%	8.9%	

For Perciformes, the fishing mortality rate (*F*) equivalent to the maximum sustainable yield (*FMSY*) is assumed to be 0.922 times the natural mortality rate (*M*) (F/FMSY ratios >1 indicate overfishing (Caddy & Mahon, 1995)). In the studied population, overfishing is clear, once *F* (0.68, or 78% of Z) was 3.5 times the *M* rate (0.17–0.35, or 22% of Z). A significant portion of captures bellow *L*_50_, *A*_50_ and *A*_100_ (38.5 cm TL, 4.5 and 11 y, respectively) (see Fig. 10, Table 3) comprises additional overfishing evidence (Froese, 2004), and may be especially relevant due to the long generation time estimated for *S. trispinosus* (20.6 years). The overfished status is more acute in coastal reefs, as juveniles represent 61.3 (gillnet) and 41.6% (spearfishing) of the landings in Caravelas, which also include very few *Lopt* and Large fish (>1.1 *L*_*opt*_) (Table 3). When catches indeed represent the age and size structure of the stock, a healthy age structure shall include 30–40% of such large fish (dubbed “mega-spawners”), in order to increase stock resilience against random events and fishing pressure (Froese, 2004; Johnson et al., 2013). Despite the more favorable indicators from offshore reefs, the Abrolhos’ parrotfish fisheries require immediate attention from managers and fishers.

Following the Red List assessment of *S. trispinosus* as an Endangered species (Ferreira et al., 2012), which was based on population decline (IUCN criterion A2d; Rosser & Mainka, 2002), a National fishing ban for all Endangered and Critically Endangered species was declared in 2014 by an Executive Order (Portaria 445/14) from the Ministry of the Environment (MMA, 2014). The National-level ban was not suggest by the scientists that assessed *S. trispinosus* status and was suspended in 2015, after a legal charge by the fishing industry backed up by the Fisheries and Aquaculture Ministry (SEAP). The Red List provides a relatively cheap and transparent framework for assessing concerning declines of exploited species, and generally agrees with fisheries metrics (Davies & Baum, 2012). However, complete fishing bans may comprise inappropriate management responses (Rice & Legacè, 2007). In 2018, MMA and SEAP settled the dispute between ministries and issued a Joint Executive Order (Portaria Interministerial 59-B/2018) with the rules and conditions for *S. trispinosus* captures. Currently, it can only be captured within the size slot of 39.0–63.0 cm TL, with spears, during the day and without air sourcing to divers. In addition, *S. trispinosus* may only be fished inside implemented multiple-use Marine Protected Areas or other types of legally established Fishery Management Areas (e.g., Acordos de Pesca). Fishes may be landed eviscerated but never in fillets, in order to allow control and monitoring. Net usage and recreational fisheries are prohibited. Compliance levels have not yet been assessed.

Management based on slot sizes may be difficult to implement in artisanal fisheries (Busilacchi et al., 2012; Freitas et al., 2014; Aschenbrenner et al., 2017), akin to most other rules established by the latest Executive Order. For instance, artisanal fisherfolks from Caravelas (Cassurubá Extractive Reserve) and other small localities with formal co-management regimes in the Abrolhos region (e.g., Corumbau Extractive Reserve) depend on nearshore catches below *L*_50_ (see Roos et al., 2016 for a similar situation in Northern Brazil). Despite such challenges and the critical state of *S. trispinosus*, it is important to recognize that complete fishing bans that depend on strict enforcement in remote and poor localities are not effective. Relatively simple fishing rules based on (often limited/data poor) scientific evidence and co-management were finally legally established for artisanal fisheries in Brazil, but their effectiveness remains to be verified by long-term monitoring. Control over commercialization and better-informed consumption are major remaining gaps, as the most valuable and often Endangered reef fishes are largely sold in larger centers or exported (Freitas et al., 2011). In addition, we remark that the novel rules add to the burden that is already being carried by Brazilian marine protected areas (MPAs), which are historically poorly planned and managed (e.g., Francini-Filho & Moura, 2008a; Francini-Filho & Moura, 2008b; Moura et al., 2013). Therefore, if long-term funding and co-management mechanisms and incentives are not introduced, fisheries management in Brazilian MPAs will not only be doomed, but may also compromise their core objectives centered on biodiversity conservation.

## Conclusion

The Brazilian-endemic and Endangered *S. trispinosus* was identified as the second largest (86.0 cm TL) and most longevous parrotfish in the Atlantic (22 years). Protogynous hermaphroditism is suggested. The species spawns year-round in South Atlantic’s largest reef complex, with reproductive peaks in February-March and August-September. The well-evident demographic partition recorded between coastal (slow-growth, ages <9 years) and offshore reefs (fast growth, ages 3–22 y) is possibly related to regional level movements, fishing effort and resource quality and availability. Due to its longevity and slow-growth, *S. trispinosus* is highly vulnerable to over-exploitation. Its contentious management history and several measures that were recently implemented (2018) are presented and discussed.

##  Supplemental Information

10.7717/peerj.7459/supp-1 Supplemental Information 1 Number of individuals per age by length (age-length key) for *Scarus trispinosus* from the Abrolhos Bank, Brazil Ages are estimated based on the first reading of the otholiths.

10.7717/peerj.7459/supp-2 Supplemental Information 2 Marginal increment rates in the otholiths of *Scarus trispinosus* from the Abrolhos Bank, Brazil

10.7717/peerj.7459/supp-3 Supplemental Information 3 Relationship between otolith weight and age of *Scarus trispinosus* (first reading) (A) relationship for all individuals in the Abrolhos sample. (A) comparison of the regression obtained for *S. trispinosus* with those from other species of *Chlorurus* and *Scarus* (data from Choat, 1996).

10.7717/peerj.7459/supp-4 Supplemental Information 4 Raw data used for backcalculated ages

10.7717/peerj.7459/supp-5 Supplemental Information 5 Raw data - Ages

10.7717/peerj.7459/supp-6 Supplemental Information 6 Raw data used for backcalculated sizes

10.7717/peerj.7459/supp-7 Supplemental Information 7 Raw data for females’ ages

10.7717/peerj.7459/supp-8 Supplemental Information 8 Raw data used to estimate VBGF for inner shelf specimens

10.7717/peerj.7459/supp-9 Supplemental Information 9 Raw data used for VBGF estimates for outer shelf specimens

10.7717/peerj.7459/supp-10 Supplemental Information 10 Raw data and growth comparisons (Kimura)

10.7717/peerj.7459/supp-11 Supplemental Information 11 Raw data and growth comparisons per sex (Kimura)

10.7717/peerj.7459/supp-12 Supplemental Information 12 Raw data and error estimates for otholith readings - APE

10.7717/peerj.7459/supp-13 Supplemental Information 13 Raw data, marginal increment

10.7717/peerj.7459/supp-14 Supplemental Information 14 Complete raw data set

## Additional Information and Declarations

### Field Study Permissions

The following information was supplied relating to field study approvals (i.e., approving body and any reference numbers): Sistema Nacional de Informação sobre Biodiversidade - SISBIO (#45738-2)

### Data Availability

The following information was supplied regarding data availability: Raw data and code are available as Supplemental Files.
