# Supplementary material for: Age, growth, reproduction and management of Southwestern Atlantic’s largest and endangered herbivorous reef fish, Scarus trispinosus Valenciennes, 1840
Source: PeerJ. 2019 Aug 30;7:e7459. doi: 10.7717/peerj.7459 (PMC6718160; doi:10.7717/peerj.7459)
Supplement: Supplemental Information 1 — Ages are estimated based on the first reading of the otholiths. [file peerj-07-7459-s001.docx]

Supplemental Information I. Number of individuals per age by length (age-length key) for *Scarus trispinosus* from the Abrolhos Bank, Brazil. Ages based on the first reading of otholiths.

| **Total Length (cm)** | **Age (years)** | | | | | | | | | | | | | | | | | | | | **Total**  **n** |
| --- | --- | --- | --- | --- | --- | --- | --- | --- | --- | --- | --- | --- | --- | --- | --- | --- | --- | --- | --- | --- | --- |
|  | 2 | 3 | 4 | 5 | 6 | 7 | 8 | 9 | 10 | 11 | 12 | 13 | 14 | 15 | 16 | 17 | 18 | 20 | 21 | 23 |  |
| (0,5] |  |  |  |  |  |  |  |  |  |  |  |  |  |  |  |  |  |  |  |  |  |
| (5,10] |  |  |  |  |  |  |  |  |  |  |  |  |  |  |  |  |  |  |  |  |  |
| (10,15] | 1 | 1 |  |  |  |  |  |  |  |  |  |  |  |  |  |  |  |  |  |  | 2 |
| (15,20] | 4 | 2 |  |  |  |  |  |  |  |  |  |  |  |  |  |  |  |  |  |  | 6 |
| (20,25] | 1 |  |  |  |  |  |  |  |  |  |  |  |  |  |  |  |  |  |  |  | 1 |
| (25,30] |  | 3 | 4 | 1 |  |  |  |  |  |  |  |  |  |  |  |  |  |  |  |  | 8 |
| (30,35] |  | 4 | 27 | 7 |  |  |  |  |  |  |  |  |  |  |  |  |  |  |  |  | 38 |
| (35,40] |  | 3 | 1 | 2 | 3 | 1 |  |  |  |  |  |  |  |  |  |  |  |  |  |  | 37 |
| (40,45] |  |  | 4 | 5 | 15 | 8 | 1 |  |  |  |  |  |  |  |  |  |  |  |  |  | 33 |
| (45,50] |  |  | 2 | 5 | 7 | 16 | 5 |  |  |  |  |  |  |  |  |  |  |  |  |  | 35 |
| (50,55] |  |  |  | 1 | 7 | 16 | 1 | 5 | 2 |  |  |  |  |  |  |  |  |  |  |  | 41 |
| (55,60] |  |  |  | 2 | 4 | 8 | 7 | 1 | 1 |  |  |  |  |  |  |  |  |  |  |  | 32 |
| (60,65] |  |  |  |  | 1 | 11 | 12 | 13 | 5 |  |  |  |  |  |  |  |  | 1 |  | 1 | 44 |
| (65,70] |  |  |  |  |  | 3 | 4 | 3 | 2 | 2 | 1 | 4 | 2 | 1 |  |  |  |  |  |  | 22 |
| (70,75] |  |  |  |  |  |  | 6 | 5 | 3 | 2 | 6 | 1 | 1 |  | 2 | 1 |  |  |  |  | 27 |
| (75,80] |  |  |  |  |  |  | 2 | 4 | 2 | 2 | 3 | 2 | 2 | 2 |  | 3 | 1 |  | 1 |  | 24 |
| (80,85] |  |  |  |  |  |  |  | 1 |  |  | 1 |  | 1 | 1 |  | 1 | 1 |  |  |  | 6 |
| (85,90] |  |  |  |  |  |  |  |  |  |  | 2 |  |  |  |  |  |  |  |  |  | 2 |
| Total | 6 | 13 | 47 | 41 | 37 | 63 | 47 | 41 | 15 | 6 | 13 | 7 | 6 | 4 | 2 | 5 | 2 | 1 | 1 | 1 | 358 |
| Mean Length (cm) | 17 | 29 | 34 | 39 | 47 | 52 | 60 | 63 | 65 | 72 | 76 | 71 | 75 | 77 | 71 | 76 | 80 | 60 | 77 | 64 | - |
| S.D. | 2.81 | 7.7 | 4.4 | 6.2 | 6.2 | 7.5 | 8.9 | 8.4 | 7.4 | 4.2 | 5.6 | 4.6 | 5.8 | 6.3 | 0 | 3.4 | 4.2 | - | - | - | - |
