# Supplementary figures and images for: Age, growth, reproduction and management of Southwestern Atlantic’s largest and endangered herbivorous reef fish, Scarus trispinosus Valenciennes, 1840
Source: PeerJ. 2019 Aug 30;7:e7459. doi: 10.7717/peerj.7459 (PMC6718160; doi:10.7717/peerj.7459)

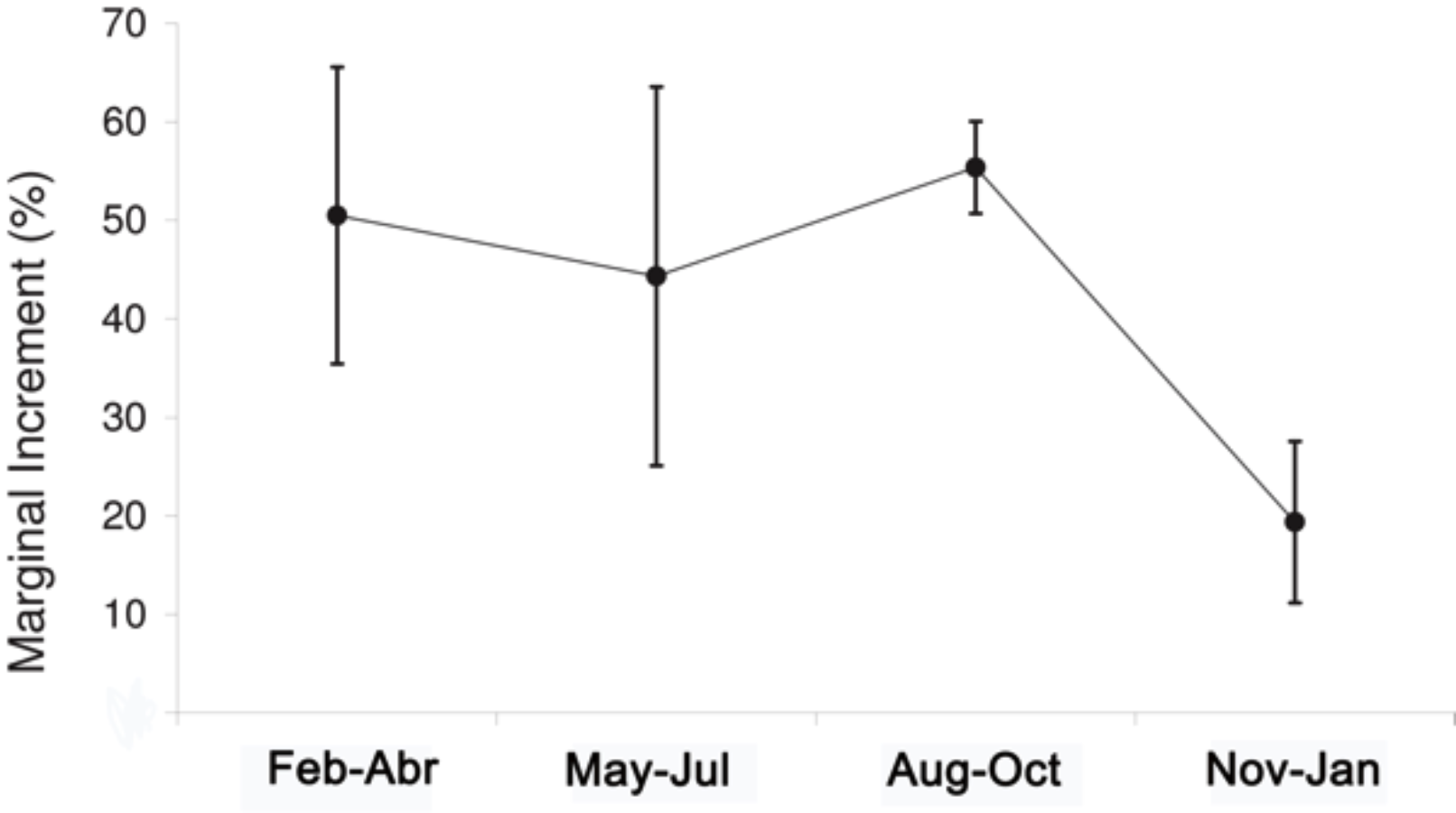

Supplement: Supplemental Information 2 [file peerj-07-7459-s002.png]

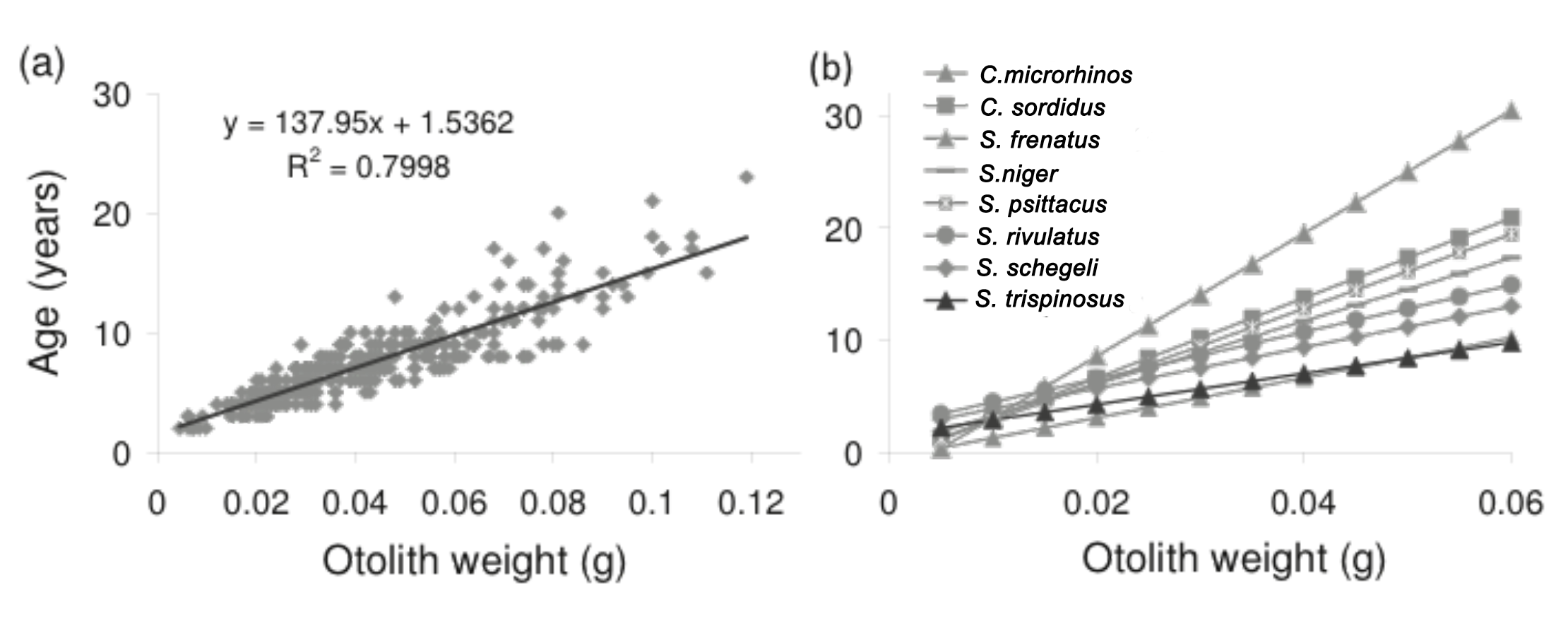

Supplement: Supplemental Information 3 — (A) relationship for all individuals in the Abrolhos sample. (A) comparison of the regression obtained for S. trispinosus with those from other species of Chlorurus and Scarus (data from Choat, 1996). [file peerj-07-7459-s003.png]
